# Supplementary material for: Coupled oscillations enable rapid temporal recalibration to audiovisual asynchrony
Source: Commun Biol. 2021 May 11;4:559. doi: 10.1038/s42003-021-02087-0 (PMC8113519; doi:10.1038/s42003-021-02087-0)
Supplement: Supplementary file 2 — Supplemental Information [file 42003_2021_2087_MOESM2_ESM.pdf]

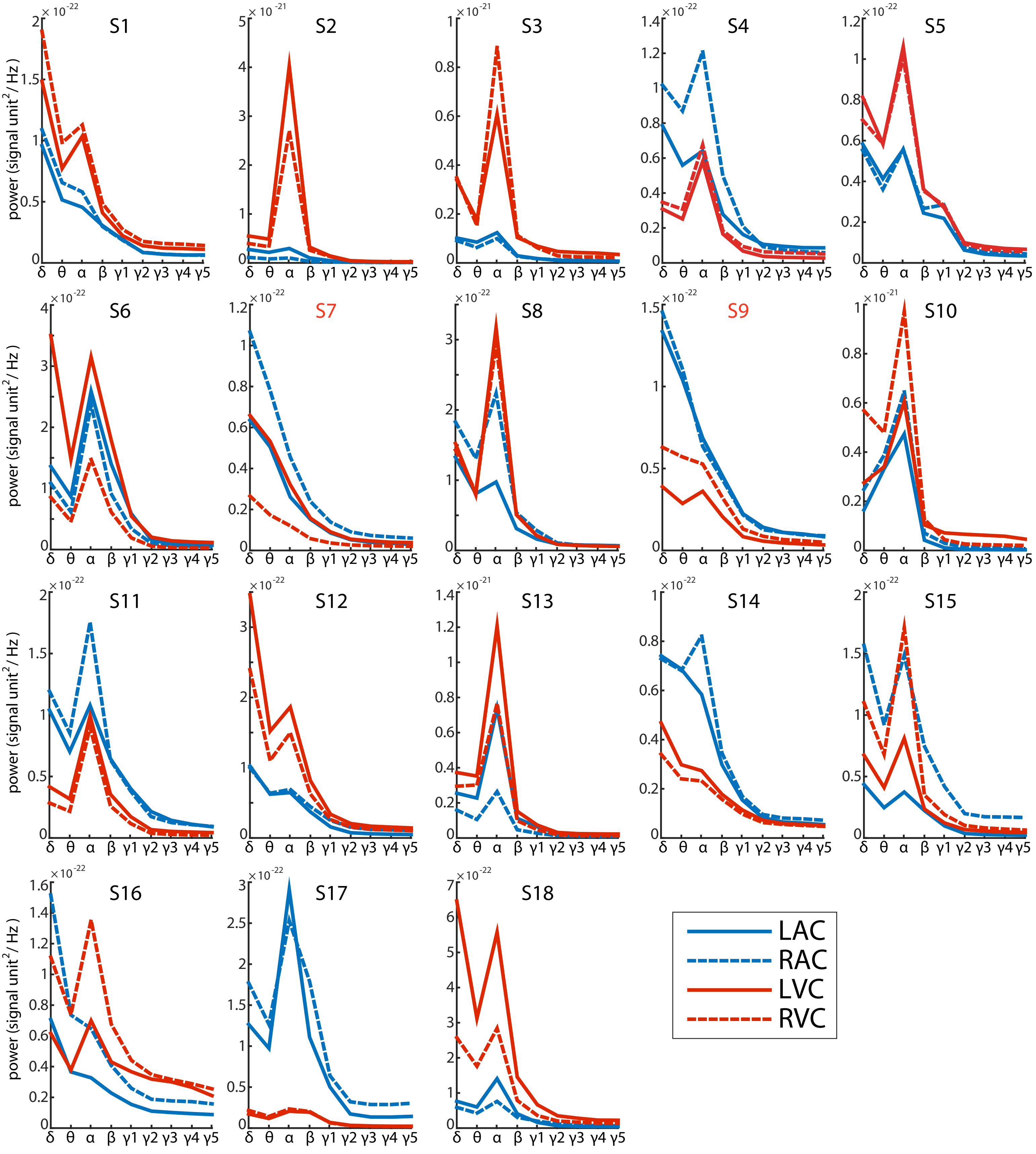

Supplementary Figure 1. Single subject power spectral densities

Single subject power spectral density computed on all trials across indicated frequency ranges for all 4 ROIs. Two subjects (S7 and S9) that did not show a clear peak in the alpha range were excluded from PAC analyses using alpha as frequency for phase.

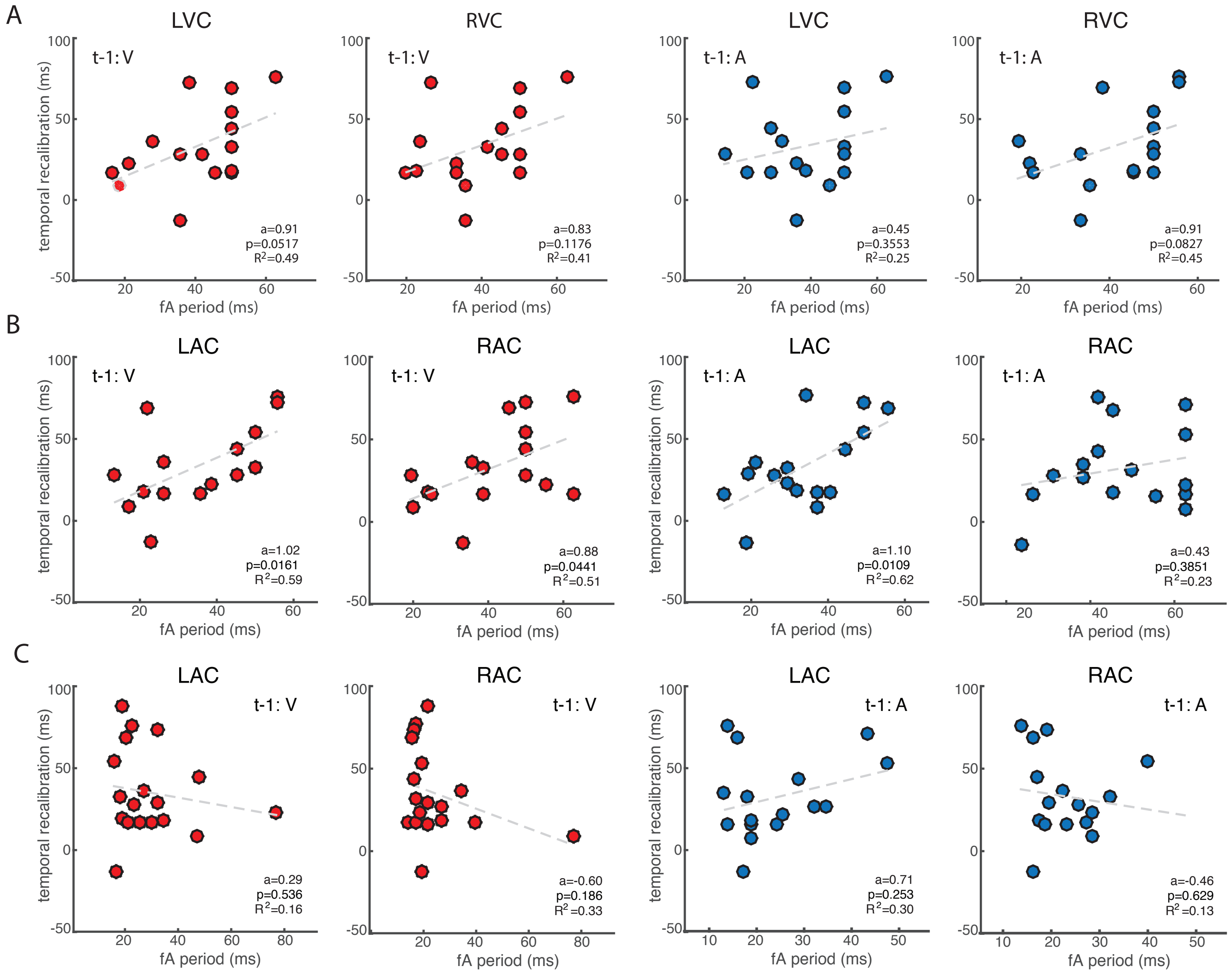

Supplementary Figure 2. Temporal recalibration as function of fA period

A) Temporal recalibration as a function of fA period measured during the pre-stimulus period in left and right visual cortex (LVC and RVC) for trials with visual lead on the previous trial (t-1:V, red dots) and those with auditory lead (t-1:A, blue dots). Dots represent single subject values. A regression line of best fit is depicted in gray along with regression coefficient a, correlation coefficient R^2, and corresponding p-value. B) The same as in A) for the post-stimulus time period (250 ms to 750 ms after stimulus onset) in left and right auditory cortex. C) The same as in B) with theta (4-7 Hz) as fP for PAC computation.

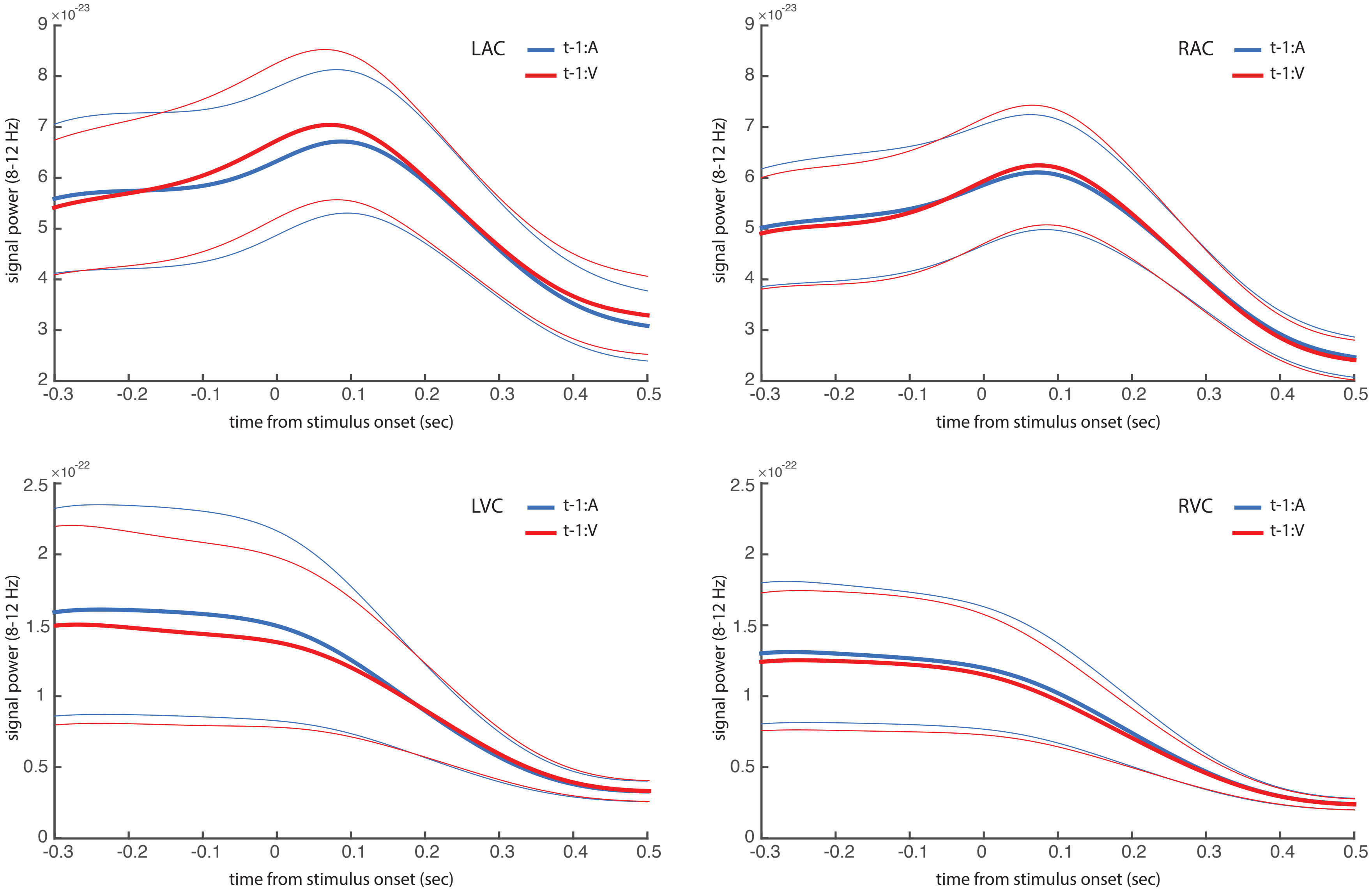

Supplementary Figure 3. Signal strength in the alpha band across the trial
In order to rule out possible confounding effects of pre-stimulus alpha power on our PAC results, we assessed signal strength in the alpha band in the ROIs. For each ROI, signal power is plotted across time from stimulus onset for t-1:A (blue) and t-1:V trials (red). Thin lines denote the corresponding +/- SEM. No significant differences were observed at the multiple comparison corrected alpha level of $p<0.0125$.

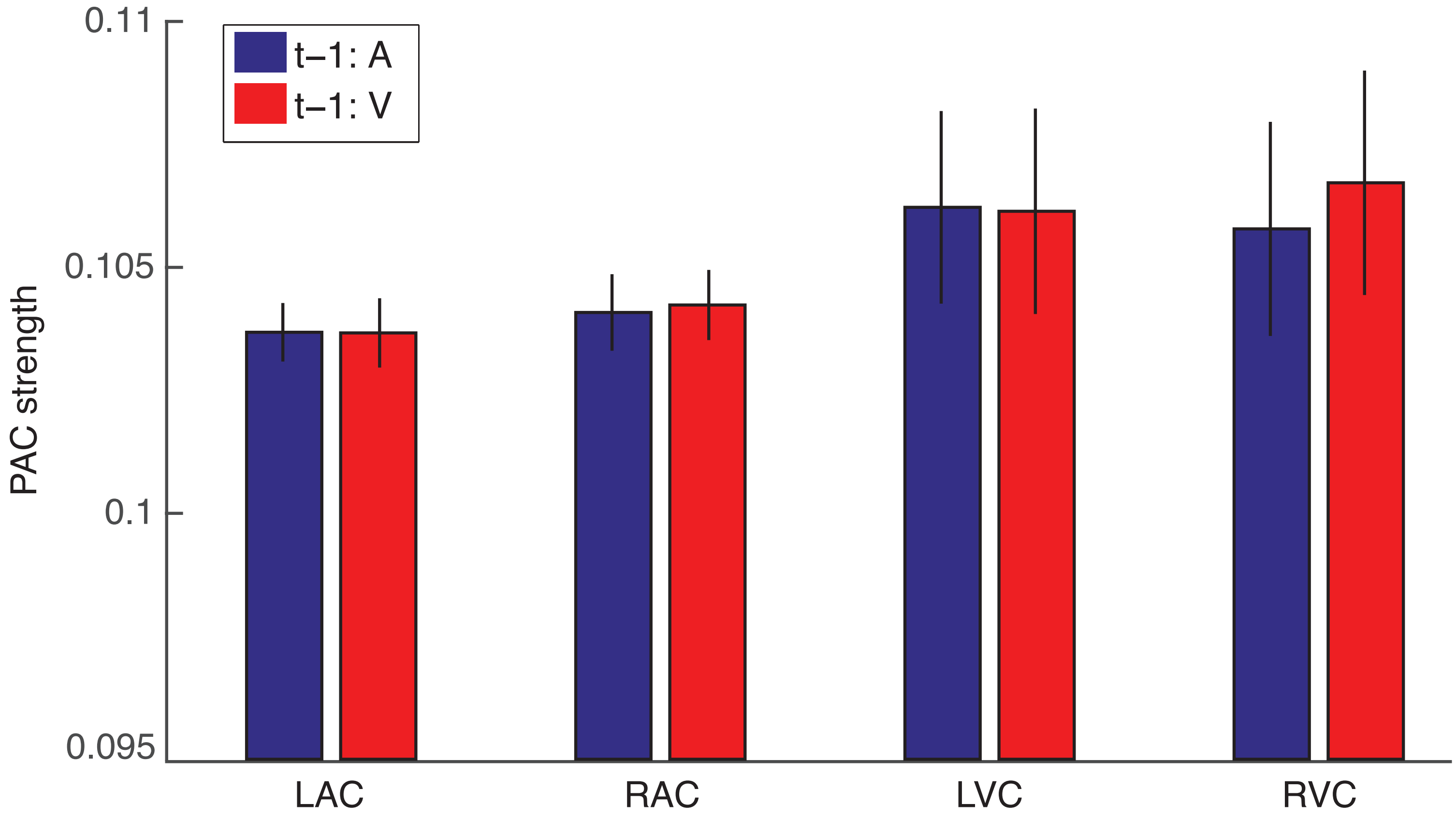

Supplementary Figure 4. PAC strength across ROIs and trial types
Average PAC strength is plotted for each ROI and trial type (blue, t-1:A; red, t-1:V). Black lines denote standard error of the mean. PAC strength was not significantly different between trial types (all $p>0.0125$).

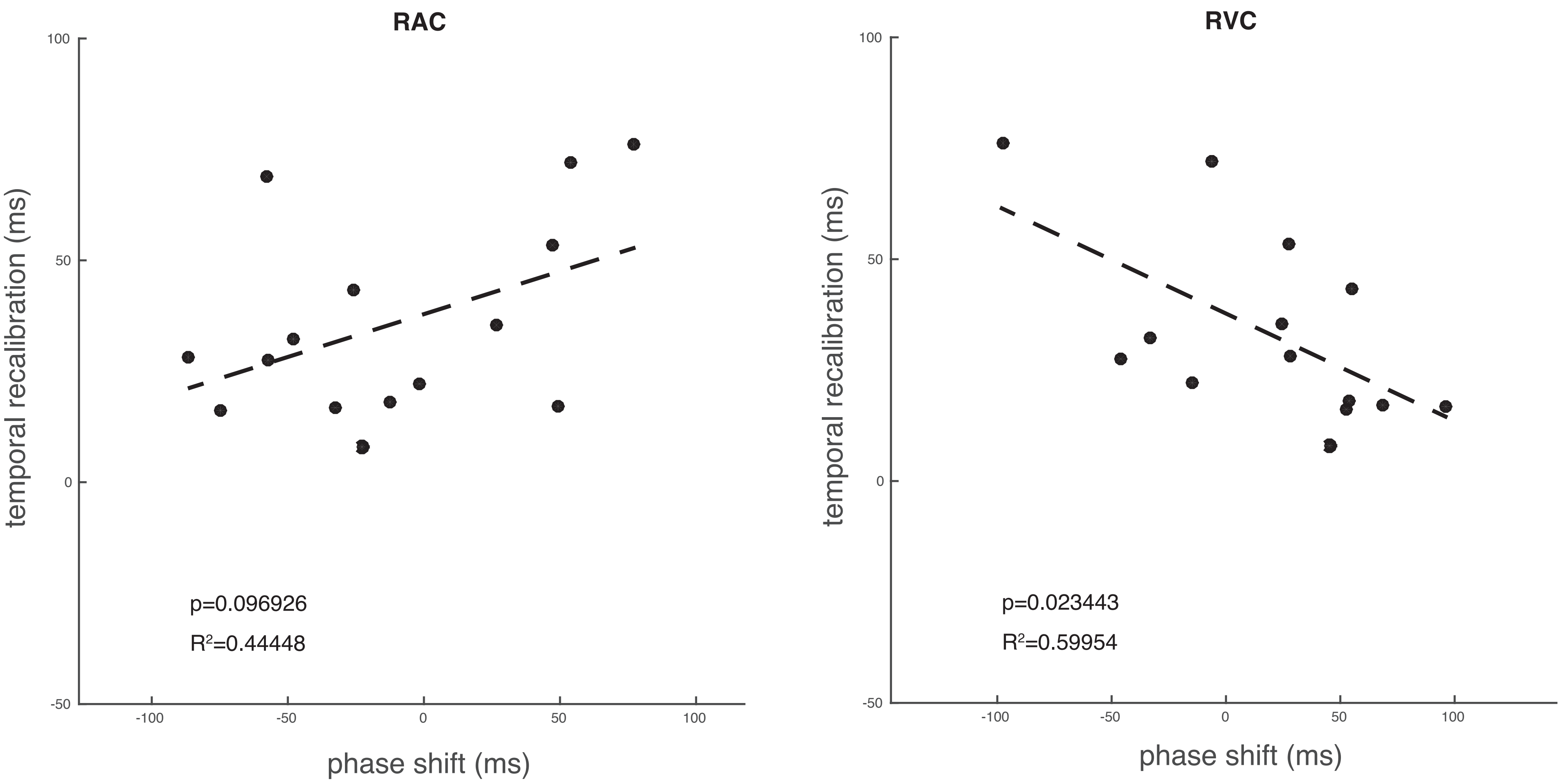

Supplementary Figure 5. Relationship between TR and phase shift

Temporal recalibration as a function of phase shift in right auditory and visual cortex. Dots represent single subject values. A regression line of best fit is depicted in black along with correlation coefficient R^2 and corresponding p-value.

Subject	% > 0				mean				std			
	LAC	RAC	LVC	RVC	LAC	RAC	LVC	RVC	LAC	RAC	LVC	RVC
1	69	63	76	78	1.10	1.16	1.83	1.82	2.07	2.86	2.41	2.55
2	73	69	80	81	1.77	1.65	2.21	2.32	2.83	2.70	2.64	3.10
3	75	68	75	77	1.57	1.61	1.98	1.76	2.31	2.73	2.77	2.41
4	70	74	81	74	1.32	1.64	1.87	1.45	2.39	2.52	2.36	2.24
5	64	67	74	65	1.48	1.37	1.65	1.07	2.83	2.70	2.48	2.88
6	79	72	79	78	2.19	2.19	1.86	2.13	2.82	3.47	2.37	2.87
7	70	65	70	71	1.66	1.21	1.72	1.42	2.69	2.86	2.91	2.50
8	71	71	75	77	1.56	1.63	1.87	1.88	2.51	2.77	2.72	2.31
9	67	68	80	76	1.43	1.47	2.02	1.62	2.92	3.06	2.50	2.25
10	70	67	71	68	1.56	1.17	1.48	1.62	2.59	2.64	2.70	2.69
11	68	70	83	79	1.68	1.96	2.21	2.06	3.30	3.15	2.52	2.74
12	64	67	73	70	1.21	1.43	1.54	1.40	3.07	2.97	2.43	2.44
13	74	69	71	72	1.89	1.56	1.44	1.81	2.72	2.73	2.31	2.78
14	69	73	75	79	1.57	1.53	1.71	1.71	2.65	2.41	2.46	2.17
15	79	73	77	75	2.38	1.55	1.70	1.67	2.87	2.47	2.43	2.33
16	74	67	70	71	1.88	1.50	1.89	1.60	2.77	2.98	3.19	2.77

Supplementary Table 1. Single subject z-scores

For all individual subjects and ROIs, percentage of z-scores above 0 is listed (left; %>0), along with mean z-scores (middle; mean) and standard deviation (right, std). The average numbers across all subjects can be found at the bottom.
