# Supplementary material for: Coupled oscillations enable rapid temporal recalibration to audiovisual asynchrony
Source: Commun Biol. 2021 May 11;4:559. doi: 10.1038/s42003-021-02087-0 (PMC8113519; doi:10.1038/s42003-021-02087-0)
Supplement: Supplementary file 3 — Reporting summary [file 42003_2021_2087_MOESM3_ESM.pdf]

nature research

Corresponding author(s): Sylvain Baillet

Last updated by author(s): 31 March 2021

Reporting Summary

Nature Research wishes to improve the reproducibility of the work that we publish. This form provides structure for consistency and transparency in reporting. For further information on Nature Research policies, see our Editorial Policies and the Editorial Policy Checklist.

Statistics

For all statistical analyses, confirm that the following items are present in the figure legend, table legend, main text, or Methods section.

n/a	Confirmed	
☐	☒	The exact sample size (*n*) for each experimental group/condition, given as a discrete number and unit of measurement
☐	☒	A statement on whether measurements were taken from distinct samples or whether the same sample was measured repeatedly
☐	☒	The statistical test(s) used AND whether they are one- or two-sided<br>*Only common tests should be described solely by name; describe more complex techniques in the Methods section.*
☐	☒	A description of all covariates tested
☐	☒	A description of any assumptions or corrections, such as tests of normality and adjustment for multiple comparisons
☐	☒	A full description of the statistical parameters including central tendency (e.g. means) or other basic estimates (e.g. regression coefficient) AND variation (e.g. standard deviation) or associated estimates of uncertainty (e.g. confidence intervals)
☐	☒	For null hypothesis testing, the test statistic (e.g. *F*, *t*, *r*) with confidence intervals, effect sizes, degrees of freedom and *P* value noted<br>*Give P values as exact values whenever suitable.*
☒	☐	For Bayesian analysis, information on the choice of priors and Markov chain Monte Carlo settings
☒	☐	For hierarchical and complex designs, identification of the appropriate level for tests and full reporting of outcomes
☐	☒	Estimates of effect sizes (e.g. Cohen's *d*, Pearson's *r*), indicating how they were calculated

Our web collection on statistics for biologists contains articles on many of the points above.

Software and code

Policy information about availability of computer code

Data collection	Brainstorm 2018
Data analysis	Brainstorm 2018, Matlab Version 9.6

For manuscripts utilizing custom algorithms or software that are central to the research but not yet described in published literature, software must be made available to editors and reviewers. We strongly encourage code deposition in a community repository (e.g. GitHub). See the Nature Research guidelines for submitting code & software for further information.

Data

Policy information about availability of data

All manuscripts must include a data availability statement. This statement should provide the following information, where applicable:

- Accession codes, unique identifiers, or web links for publicly available datasets
- A list of figures that have associated raw data
- A description of any restrictions on data availability

The dataset generated and analyzed during the current study are available from the corresponding author upon reasonable request.

Field-specific reporting

Life sciences study design

All studies must disclose on these points even when the disclosure is negative.

Sample size	We considered a sample size (n=20) that is typical of hypothesis-driven MEG studies. The sample size was decided prior to collecting data and data analysis was initiated after all data samples were collected.
Data exclusions	2 subjects were excluded because of preprocessing issues (strong artifacts and faulty cortex segmentation). 2 out of the remaining 18 participants were excluded from PAC analyses because a well-defined spectral peak necessary for the meaningful estimation of PAC was absent.
Replication	We haven't addressed reproducibility. This is an original study, presenting a new set of phenomena.
Randomization	Different trial types were presented at random; the button assignments were randomized from one block to the next.
Blinding	Blinding was not relevant in our study; we used an event-related design with randomized trial presentation.

Reporting for specific materials, systems and methods

We require information from authors about some types of materials, experimental systems and methods used in many studies. Here, indicate whether each material, system or method listed is relevant to your study. If you are not sure if a list item applies to your research, read the appropriate section before selecting a response.

Materials & experimental systems

n/a	Involved in the study
☒	☐ Antibodies
☒	☐ Eukaryotic cell lines
☒	☐ Palaeontology and archaeology
☒	☐ Animals and other organisms
☐	☒ Human research participants
☒	☐ Clinical data
☒	☐ Dual use research of concern

Methods

n/a	Involved in the study
☒	☐ ChIP-seq
☒	☐ Flow cytometry
☒	☐ MRI-based neuroimaging

Human research participants

Policy information about studies involving human research participants

Population characteristics	8 female, 10 male; between ages 20 and 45; healthy.
Recruitment	Recruitment mostly through a subject database.
Ethics oversight	Montreal Neurological Institute Research Ethics Board

Note that full information on the approval of the study protocol must also be provided in the manuscript.
